# A Fine-Scale and Minimally Invasive Marking Method for Use with Conventional Tungsten Microelectrodes

**DOI:** 10.1523/ENEURO.0141-23.2023

**Published:** 2023-09-20

**Authors:** Tatsuya Oikawa, Kento Nomura, Toshimitsu Hara, Kowa Koida

**Affiliations:** 1 Department of Computer Engineering, Toyohashi University of Technology, Aichi 441-8580, Japan; 2 Institute for Research on Next-generation Semiconductor and Sensing Science, Toyohashi University of Technology, Aichi 441-8580, Japan

**Keywords:** electrode, electrophysiology, *in vivo*, marking, unit recording

## Abstract

In neurophysiology, achieving precise correlation between physiological responses and anatomic structures is a significant challenge. Therefore, the accuracy of the electrode marking method is crucial. In this study, we describe a tungsten-deposition method, in which tungsten oxide is generated by applying biphasic current pulses to conventional tungsten electrodes. The electrical current used was 40–50 μA, which is similar to that used in electrical microstimulation experiments. The size of the markings ranged from 10 to 100 μm, corresponding to the size of the electrode tip, which is smaller than that of existing marking methods. Despite the small size of the markings, detection is easy as the marking appears in bright red under dark-field observation after Nissl staining. This marking technique resulted in low tissue damage and was maintained *in vivo* for at least two years. The feasibility of this method was tested in mouse and macaque brains.

## Visual Abstract

**Figure:**
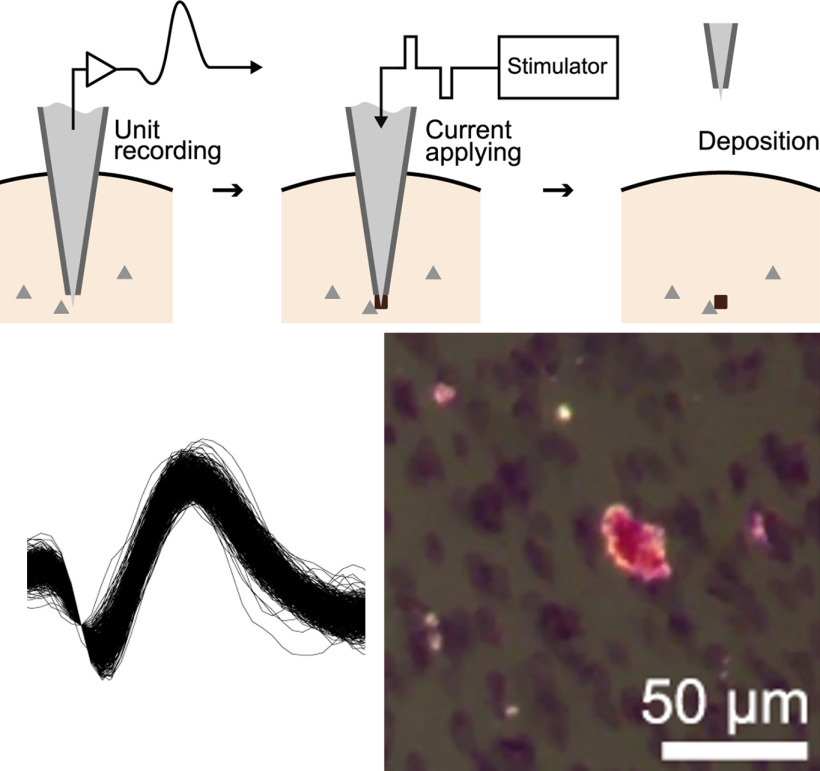

## Significance Statement

A new marking method was developed to identify the recording site for conventional tungsten microelectrodes. Applying biphasic pulse current oxidizes the tip of the tungsten electrode, then the oxides are deposited around the recording site to form a marking. The markings were smaller than those formed in existing methods, caused less damage to the brain tissue, remained for at least two years, and were easy to identify by dark-field observation after cresyl violet staining.

## Introduction

Mapping neuronal activity to anatomic structures is a fundamental issue in the field of neuroscience. For over 50 years, *in vivo* extracellular recordings using microelectrodes have been used to understand physiological activities and functional structures at the single-neuron level. Although modern imaging techniques have made significant advances, they still have limitations in terms of spatiotemporal resolution (Ogawa et al., 1990a, b; Logothetis et al., 2001; Logothetis, 2008) and imaging depth (Kobat et al., 2009; Horton et al., 2013; Yildirim et al., 2019; Takasaki et al., 2020). As a result, electrophysiological methods have remained crucial for recording activity in deep brain structures, particularly in large animals. Multielectrode arrays enable the recording of multiple neurons simultaneously from deep brain regions (Glimcher et al., 2001; Hansen et al., 2011; Jun et al., 2017; O’Keefe and Recce, 1993; Recce and O’Keefe, 1989; Steinmetz et al., 2021; Wilson and McNaughton, 1993), but the relatively large size of the electrodes can cause significant brain damage. Furthermore, the equipment required for these methods is expensive. In contrast, single-channel electrodes generally possess a small diameter, enabling the recording of single-unit activities from deep brain regions with less damage (Szarowski et al., 2003) and at a lower cost. Therefore, single-unit recordings using conventional electrodes are useful for investigating neuronal function in the deep brain.

In order to establish a correlation between physiological responses and anatomic structures, it is necessary to accurately identify the recording site. In single-unit recordings, the position of the electrode tip is approximately determined using stereotaxic coordinates to insert the electrode at the desired recording position (Horsley and Clarke, 1908; Saunders et al., 1990; Asahi et al., 2003). Ideally, this method would provide the exact location of the electrode tip; however, in practice, it is challenging to accurately estimate the recording site because of deformation of the electrode and brain tissue. This estimation error is particularly significant for recordings from deep brain regions. To address this issue, several subsidiary visualization methods have been developed, including ultrasonic echo (Collier et al., 1980; Tokuno et al., 2000; Glimcher et al., 2001), x-ray and computed tomography (CT; Aggleton and Passingham, 1981; Nahm et al., 1994; Cox et al., 2008), as well as magnetic resonance imaging (MRI; Jog et al., 2002; Martínez Santiesteban et al., 2006; Tammer et al., 2006; Matsui et al., 2007), to visualize the electrode tip *in vivo*. However, these methods have limitations in spatial resolution at the submillimeter scale and low tissue contrast. Therefore, mapping the electrode site to the brain anatomy using these *in vivo* methods is limited.

Various marking methods have been developed to accurately identify the recording sites using postmortem histology. Among these methods, electrolytic microlesion (Hubel and Wiesel, 1962) has been the most widely used technique for over 60 years. This technique involves passing dynamic current (DC) from the tip of the electrode to damage the tissue around the tip. The resulting damage and gliosis can be confirmed by staining the tissue. Another commonly used technique involves coating the electrode with a fluorescent dye and observing the penetration path with fluorescence (Snodderly and Gur, 1995; DiCarlo et al., 1996; Naselaris et al., 2005). This technique is easy to implement and does not cause damage to the brain tissue. Stainless steel or elgiloy electrodes can be marked by depositing iron through the application of anode current, which can then be visualized with additional staining (Adrian and Moruzzi, 1939; Hess, 1939; Marshall, 1940; Brown and Tasaki, 1961; Suzuki and Azuma, 1987). There are also several methods that use nonmetallic electrodes. For example, intracellular recording with glass-tube electrodes can be marked by injecting dye (ion implantation; Thomas and Wilson, 1965; Stretton and Kravitz, 1968; Lee et al., 1969), and juxtacellular recordings can be marked by intracellular dye injection (Pinault, 1996).

All of the methods described above have certain limitations, including low spatial resolution, high damage, and short survival times. The existing marking methods typically have a width of around 100 μm, which is insufficient to accurately localize the site smaller than 100 μm, such as suborganizations within small nuclei. Although the size of lesion marking and iron ion marking could be reduced by using a smaller current, it may also lead to diffusion or disappearance of the markings over time. Both lesion marking and metal ion deposition techniques involve the application of electric current, which can cause tissue damage and obscure anatomic structures. The damage also limits further recording in the vicinity of the marking. Using biphasic currents instead of DC during iron ion markings could reduce damage; however, there is still a trade-off between the persistence of the marking and their size (Fung et al., 1998). Intracellular dye injection itself does not cause damage, but the thick glass tube electrodes used in the technique can lead to significant tissue damage during penetration. Finally, the use of neurobiotin dye in juxtacellular recording is limited by its short survival time of up to 48 h. This poses a significant limitation for long-term chronic experiments, particularly in large animals, where experiments can last for months or years.

None of the existing marking methods meet the requirements of spatial resolution, damage, and survival time simultaneously. These requirements are particularly important for mapping single-unit activity with the small structures in the deep brain, where subcortical nuclei and ciliary bodies are small and often have substructures in the order of 100 μm, such as layers of the lateral geniculate nucleus and superior colliculus (Kwan et al., 2019; Turner et al., 2020).

In this study, we describe a tungsten-deposition method, where tungsten oxide is generated by the application of biphasic current to a conventional tungsten electrode. This method is similar to one previously described by Pabst (Pabst, 1973). However, the previous method had three issues. First, the anode reaction stops when the electrode tip is covered with an insulating oxide film because of the application of DC. The insulating film prevents further reaction and generates insufficient amounts of tungsten oxide, leading to uncertain marking unless DC polarity is repeatedly switched. Second, the use of DC causes damage to brain tissue, similar to the lesion marking technique. Third, the deposited tungsten cannot be distinguished from artifacts without additional staining using a strong acid. This reduction treatment makes it difficult to perform in parallel with existing cell staining techniques that are sensitive to the pH of the solutions.

**Figure 1.**
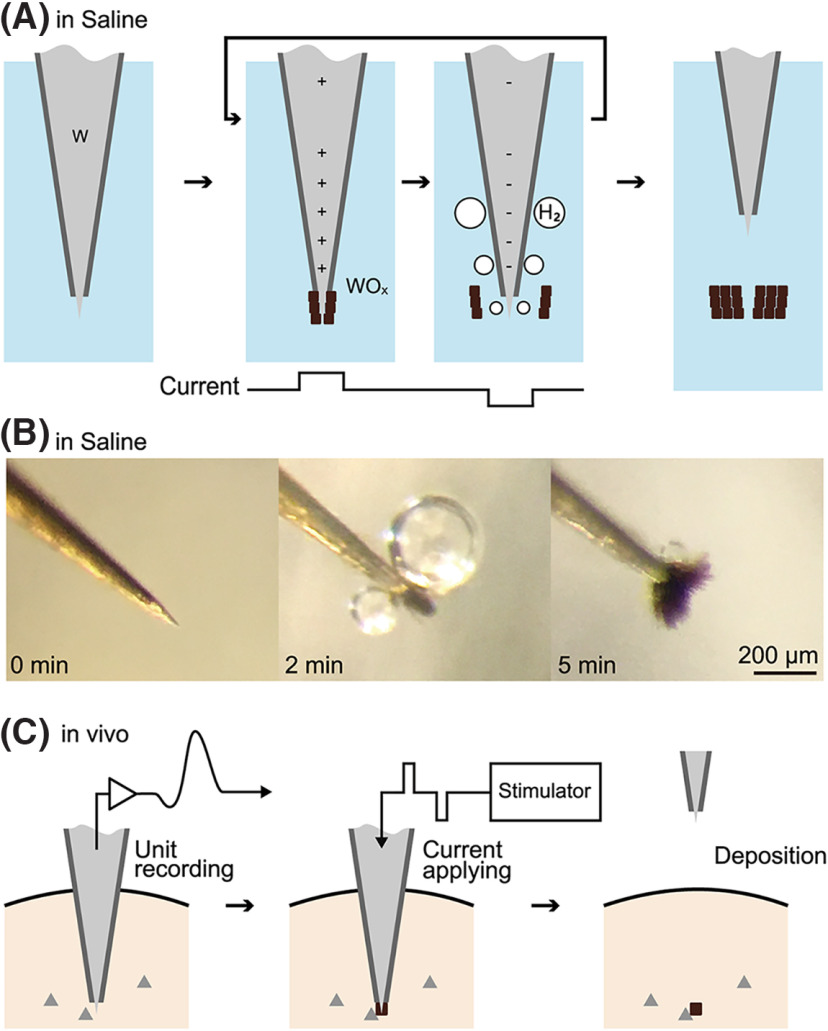
The marking method. ***A***, Schematic diagram outlining the generation of tungsten oxide. By subjecting a tungsten electrode (W) to biphasic current in a saline solution, an anode reaction leads to the production of tungsten oxide (WOx), while a cathode reaction produces hydrogen bubbles (H_2_). These reactions repeat for a certain duration until an adequate amount of tungsten oxide deposits around the tip. ***B***, The photographs illustrate the electrode tip’s appearance on application of bipolar current. ***C***, Experimental protocol for *in vivo* implementation of the marking technique.

To address these issues, we employed a biphasic current instead of DC. Biphasic current is less likely to be impeded by the oxide films and is considered to be safer for the tissue than DC (Salzman et al., 1992; Tehovnik, 1996; Cohen and Newsome, 2004; Clark et al., 2011). A schematic diagram of this method is shown in Figure 1*A*. When the biphasic pulse is applied, an insoluble oxide film is generated via the anodic reaction, then the oxide is detached from the electrode through bubbles created by the cathodic reaction (Robblee and Sweeney, 1996). By repeating these reactions for several minutes, an amount of oxide can be produced (Fig. 1*B*) and dispersed around the electrode tip (in this image, some oxide has adhered to the electrode tip).

In addition, we conducted *in vivo* experiments to demonstrate the applicability of our method. Figure 1*C* illustrates the experimental design, which involved conducting single-unit recordings of the deep brain structure in animals and marking the recording site using biphasic current application. The results revealed that our method produced marking of several tens of micrometers in size with minimal tissue damage. Notably, successful markings were obtained in the brain of a macaque monkey, and they persisted in the living animal for more than two years.

## Materials and Methods

### Experimental models and subject details

The experimental procedures using mice were conducted in accordance with the national and institutional guidelines and were approved by the Animal Care and Use Committee of Toyohashi University of Technology (2020-1). A total of three wild-type mice (C57BL/6J, one male and two females, 22.6–26.4 g body weight) aged at least 10 weeks old were used. The animals were housed in a cage at room temperature (20–24°C) with a 12/12 h light/dark cycle and fed daily.

The experimental procedures using macaque monkey were conducted in accordance with the National Institutes of Health *Guide for the Care and Use of Laboratory Animals* (1996) and were approved by the Institutional Animal Experimentation Committee of the National Institute of Physiologic Sciences, Okazaki. One Japanese macaque (*Macaca fuscata*, male, 7.0 kg body weight) was used for the experiments. The animal was housed in a cage at room temperature (20°C–24°C) with 12/12 h light/dark cycle and fed daily.

### Electrophysiology (mouse)

The mice were anesthetized using a combination of medetomidine (0.75 mg/kg), midazolam (4 mg/kg), and butorphanol (5 mg/kg) via intraperitoneal injection. During recording and marking, the mice were immobilized with a stereotaxic instrument (Narishige). Tungsten microelectrodes (3.0–4.0 MΩ, UEWLEGLMFNNH, FHC for two mice, 1.2–1.4 MΩ, UEWLEGLM, FHC for one mouse) were stereotaxically mounted and vertically penetrated into the right hemisphere of the brain via the fenestra of the cranium and dura mater (1–3 mm caudal and 1–3 mm lateral to the bregma). Before insertion, the electrode tip was coated with fluorescent dye (DiI; Sigma-Aldrich; DiCarlo et al., 1996). The electrodes were inserted at the depth of the thalamus until a well-isolated single unit was obtained. For a reference electrode, a stainless screw electrode was inserted into the skull of the left hemisphere.

Recordings were performed using a headstage (SH16, Tucker-Davis Technologies) with filters (0.35 Hz for low cutoff and 7.5 kHz for high cutoff). The signals were routed to a preamplifier/digitizer (PZ2, Tucker-Davis Technologies) and acquired with a digital signal-processing module (RZ2, Tucker-Davis Technologies). Digital data were stored on a PC with a sampling frequency of 25 kHz. Spike activity was detected offline from the filtered signals (0.5–3 kHz, second-order Butterworth filter) by thresholding in MATLAB (MathWorks). To isolate unit activities, the triggered signals were processed with a window discriminator.

Markings were made by applying an electric current generated by the electronic stimulator (SEN-3401, Nihon Koden), with the current being controlled by the isolator (SS-203J, Nihon Koden). Biphasic current pulses (40 μA, 200 Hz, anodal leading, pulse width = 0.5 ms for both anode and cathode pulses, pulse interval = 0 ms) were applied via the electrode for 180 s. Following current application, the electrode was gently removed.

### Histology (mouse)

After one week of *in vivo* recording, mice were deeply anesthetized through intraperitoneal injection of urethane (1 g/kg). Then, the mice were transcardially perfused with 0.1 m PBS [PBS (−)], and fixed by perfusing 4% paraformaldehyde (PFA) in 0.1 m phosphate buffer (PB). The brains were postfixed with 4% PFA overnight at 4°C and then placed in a solution of PB with sucrose at increasing concentrations of 10%, 20%, and 30% until they sank. Subsequently, the brain was sliced coronally into 100-μm thicknesses in a cryostat and stained with cresyl violet. The slices were then examined under an optical microscope (CX21, Olympus), with the ability to switch between bright-field and dark-field microscopy using a homemade patch stop.

### Electrophysiology (macaque)

The surgical procedure for the monkey has been described previously (Matsumora et al., 2008; Koida and Komatsu, 2023). In brief, the monkey’s head was fixed by a head holder, and the eye position was recorded using a video-based monitoring system (120 Hz, ISCAN). The recording chamber was placed on the skull, where an electrode could be inserted vertically into a region of interest in the ventral surface of the inferior temporal cortex (IT). This was guided by landmarks on MRI obtained before the surgery. Recording positions were determined by comparing the depth profile of neuron activities during each penetration with the MRI images, as well as the tip of the electrode identified in x-ray photographs.

Neural recordings and microstimulations were made using electrodes inserted through a grid of evenly spaced holes at 1 mm intervals (Crist et al., 1988) over a wide area of the ventral surface of the IT. The experiment was performed when the electrode was advanced >200 μm from the entry depth of the deep layer. After the unit recording, marking (microstimulation) was performed through the same electrode.

The microstimulations were performed by applying an electric current using an electronic stimulator (SEN-7203, Nihon Koden), with the current controlled by the isolator (SS-203J, Nihon Koden). Biphasic current pulses (20 or 50 μA, 200 Hz, anodal leading, pulse width = 0.2 ms for both anode and cathode pulses, pulse interval = 0 ms) were intermittently applied via the electrode for a total of 300 s. Following current application, the electrode was gently removed.

### Histology (macaque)

After two years of *in vivo* recording, the monkey was deeply anesthetized by intravenous injection of pentobarbital sodium (80 mg/kg), which was transcardially perfused with PB and fixed by perfusing 4% PFA in 0.1 m PB. The histologic protocol and microscopy were same as those used in the mouse experiments.

## Results

### Mouse experiment

**Figure 2.**
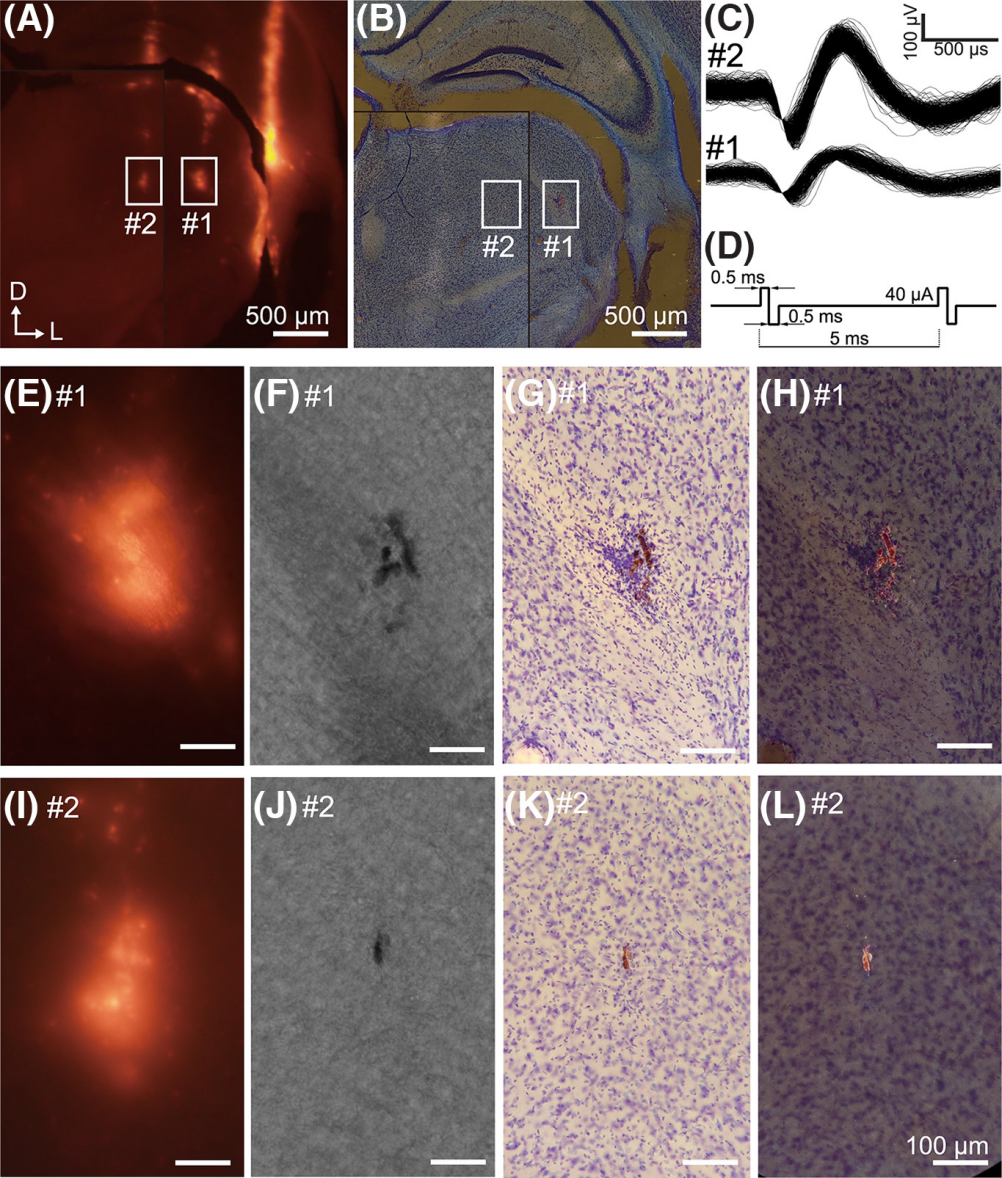
Marking experiment in a mouse. ***A***, Fluorescent image of the coronal slice of a mouse brain. Dye traces are observable as three electrode penetrations. Two consecutive slices were merged and separated by the border of the black line. ***B***, Dark-field image of the same field seen in ***A***. ***C***, Spike waveforms recorded at the site of markings indicated in ***A***. ***D***, Temporal waveform of the applied current. ***E–H***, Magnified views of the markings shown in box #1. ***E***, Fluorescent image. ***F***, Bright-field image (unstained). ***G***, Bright-field image (Nissl stained). ***H***, Dark-field image (Nissl stained). ***I–L***, Magnified views of the markings shown in box #2. Imaging method employed in each figure was the same as in ***E–H***. Scale bars in ***E–L*** indicate 100 μm.

Fluorescence (DiI)-coated tungsten electrodes were vertically inserted into the mouse brain (Fig. 2*A*,*B*). Electrophysiological recordings were taken from the thalamus (Fig. 2*C*), followed by the application of biphasic microcurrent (40-μA biphasic pulses, 180 s; Fig. 2*D*) for marking purposes (for details, see Materials and Methods). Three electrodes were penetrated, each separated by a lateral distance of 500 μm. After the current application, the electrode tips were slightly rounded, and their impedance was reduced. Two weeks after marking, perfusion was performed, followed by coronal slicing of the brain.

Before staining, fluorescence images of the slices revealed three parallel tracks of penetration (Fig. 2*A*), which were spaced at ∼500-μm intervals. At the deepest site of each track, except the lateral one, a strong fluorescent spot appeared (indicated by #1 and #2), which was located ∼3000 μm below the brain surface, consistent with the depth of penetration for recording and marking. Thus, the fluorescent spots were likely the results of electrical pulses that induced the release of DiI from the electrodes (DiCarlo et al., 1996). The other fluorescent track on the lateral track was ∼1000 μm deeper than the inner two, which is consistent with the electrode penetrations that were 1000 μm deeper on the manipulator than the inner two. No bright spot at the deepest site was found on this track.

Markings of tungsten oxide were successfully detected in two of the three tracks located at the deepest site of the track (Fig. 2*B*). These markings were identified on two consecutive slices and were subsequently merged into a single figure. No markings were detected on the lateral track. In bright-field observation, the markings appeared as black particles within the unstained slice (Fig. 2*F*,*J*). After subsequent Nissl staining, the markings appeared as brown particles (Fig. 2*G*,*K*). Marking #1 comprised several elongated particles, with the total size of the marking being 54 × 174 μm (Fig. 2*F–H*). This marking was concomitant with a slight lesion (Fig. 2*G*). Conversely, marking #2 appeared to comprise a single elongated particle, with a total size of 21 × 53 μm. No lesions were observed around this marking (Fig. 2*K*).

Bright red markings were prominent in dark-field microscopy (where illumination originates from the periphery, then the scattered light from the sample forms the image, Fig. 2*B*,*H*,*L*). These bright features were exclusively detected at the marking sites and were absent from the black, unidentified grains sometimes encountered in the sections. Therefore, the bright red markings could be attributed to the reflecting light from the surface of tungsten oxide. Comparing images between bright-field and dark-field microscopy could aid in distinguishing the marking from artifacts such as dust. Additionally, dark-field microscopy enabled fast exploration of markings with low-magnification and a large field of view, which facilitated prompt detection of tiny markings (Fig. 2*B*).

The accuracy of the positioning of the marking was verified by the good overlap with the fluorescent spots and the penetration depth. The fluorescent spots indicated the deepest point of the penetration, which aligned with the markings, since the electrode was not advanced beyond the depth of the electrical stimulation sites. Moreover, the location of the marking corresponded to the lesion site probably caused by the electrical pulses in marking #1. These coincidences suggest that the sites of the marking matched the sites where electrical pulses were applied. The same experiments were replicated on two other mice and a total of nine markings, including the initial experiment. Among these, we successfully identified six markings (66%). The size of markings ranged from 12 to 194 μm in the major diameter, 10–54 μm in the minor diameter. Therefore, our marking technique is feasible *in vivo* and exhibits good positional accuracy.

### Macaque experiment

The application of biphasic pulses, commonly known as microstimulation, is widely used for stimulating neurons to investigate behavioral effects (Lilly et al., 1955; Murasugi et al., 1993; Tehovnik, 1996; Cohen and Newsome, 2004; Murphey and Maunsell, 2007; Clark et al., 2011; Cicmil and Krug, 2015). The electrical currents used in these stimulation experiments (10–50 μA) were equivalent to the current used in our biphasic pulses marking method (40 μA). Therefore, we hypothesized that tungsten oxide would remain in the brains where electrical microstimulation was applied. To test this hypothesis, we investigated the brain of a macaque monkey in which microstimulation was applied for the purpose of modulating neuronal activities (Koida and Komatsu, 2023).

**Figure 3.**
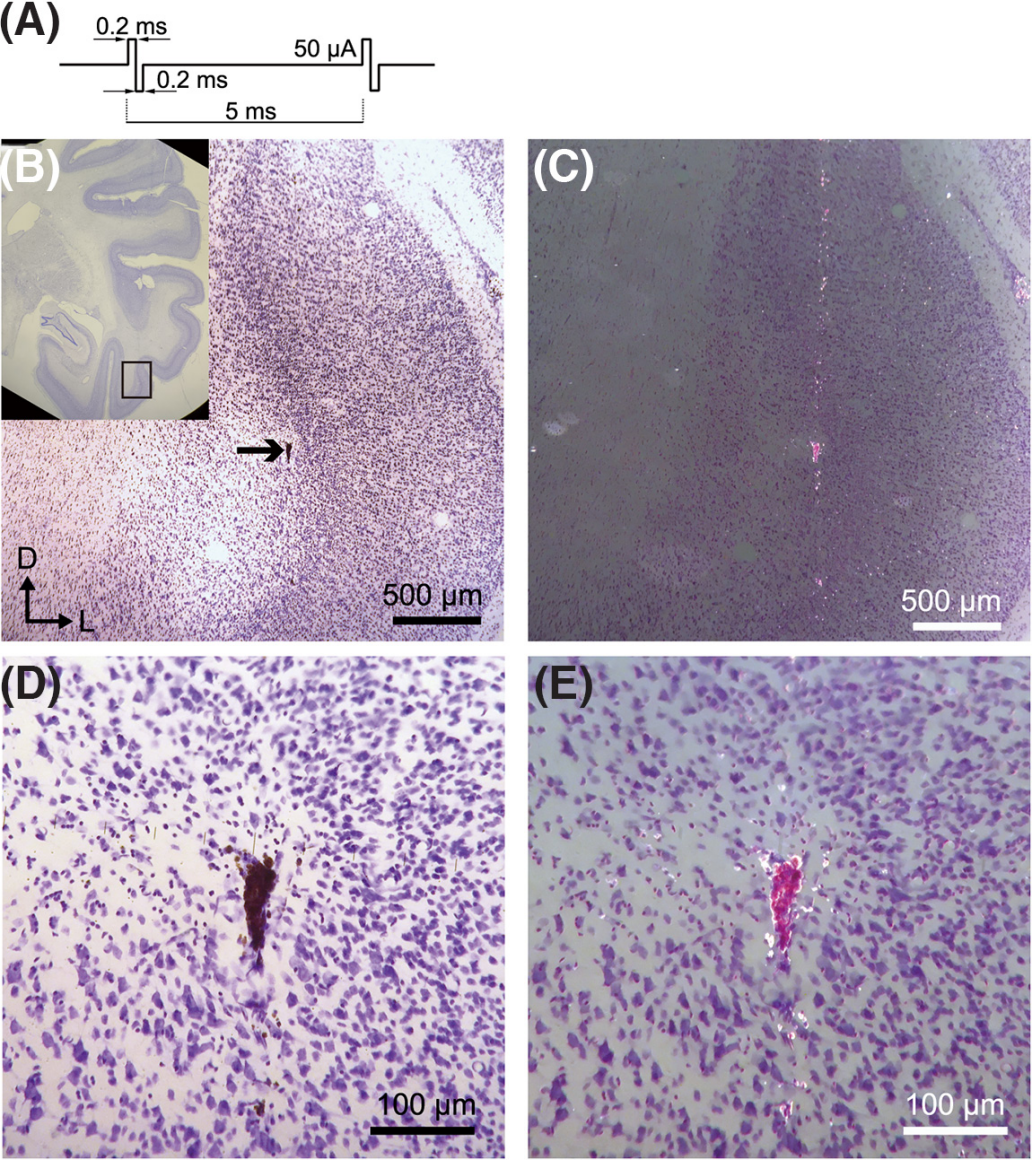
Marking on a macaque monkey. Nissl-stained coronal section is shown. ***A***, Temporal waveform of the applied current. ***B***, Deposition of tungsten oxide was found at the inferior temporal cortex (indicated by arrow). The inset displays a macroscopic view of the slice. The square denotes the region of the main panel. ***C***, Dark-field image of ***A***. ***D***, Magnified view of ***A***. ***E***, Magnified view of ***B***.

Tungsten electrodes were inserted vertically into the inferior temporal cortex of the macaque monkey, after which electrical microstimulation (biphasic, 20 or 50 μA) was applied periodically (for details, see Materials and Methods). At least two years after the microstimulation, perfusion, and slice preparation were performed. We analyzed these slices after 10 years had elapsed since the perfusion. The marking of tungsten oxide was present in these slices (Fig. 3). The markings were visible as dark, arrowhead-shaped grains in the bright-field image (Fig. 3*B*), while in the dark-field image, the markings appeared in bright red (Fig. 3*C*). The markings were 89 × 38 μm in size (Fig. 3*D*,*E*). The cells around the marking were secure, although there was some deformation of the cells. We also observed bright red powders distributed along a vertical straight line passing through the markings (Fig. 3*C*). These markings may have resulted from the electrical microstimulation, while the powdery lines were likely the result of the scattering of the tungsten oxide by the movement of the electrode after microstimulation. This was confirmed by the itinerary of electrode penetration, which involved an additional advancement of 1500 μm deeper after the microstimulation and removal.

Electrical microstimulation of 50 μA was performed at 22 sites for this hemisphere via different electrodes. Among those microstimulations, 14 markings were found at approximately corresponding sites. The size of markings ranged from 25 to 149 μm in the major diameter, 16–59 μm in the minor diameter, except for the powdery line. The low probability (64%) of detection may be because of the fact that only one of the two consecutive slices was preserved. In contrast, microstimulation of 20 μA, which was performed on the other hemisphere, yielded only minor powdery oxide, and virtually no marking was observed around the stimulation sites. Our marking method is therefore applicable to the monkey brain if a certain amount of current is applied, and the marking is preserved for more than two years *in vivo*.

## Discussion

We have described a novel marking technique that applies biphasic pulsed current via conventional tungsten electrodes. The application of the current results in the corrosion of the electrodes, which is commonly referred to as electroetching (or electropolishing; Hubel, 1957; Levick, 1972; Sugiyama et al., 1994; Szostak et al., 2017). The by-product of the electroetching, tungsten oxide, is then deposited around the electrode tip and forms a marking. The technique was successfully validated in both the mouse and the macaque monkey, demonstrating high precision, low invasiveness, and persistence *in vivo* for over two years. These results surpass the existing marking techniques in terms of localizability, identifiability, invasiveness, persistence, and cost-effectiveness.

**Figure 4.**
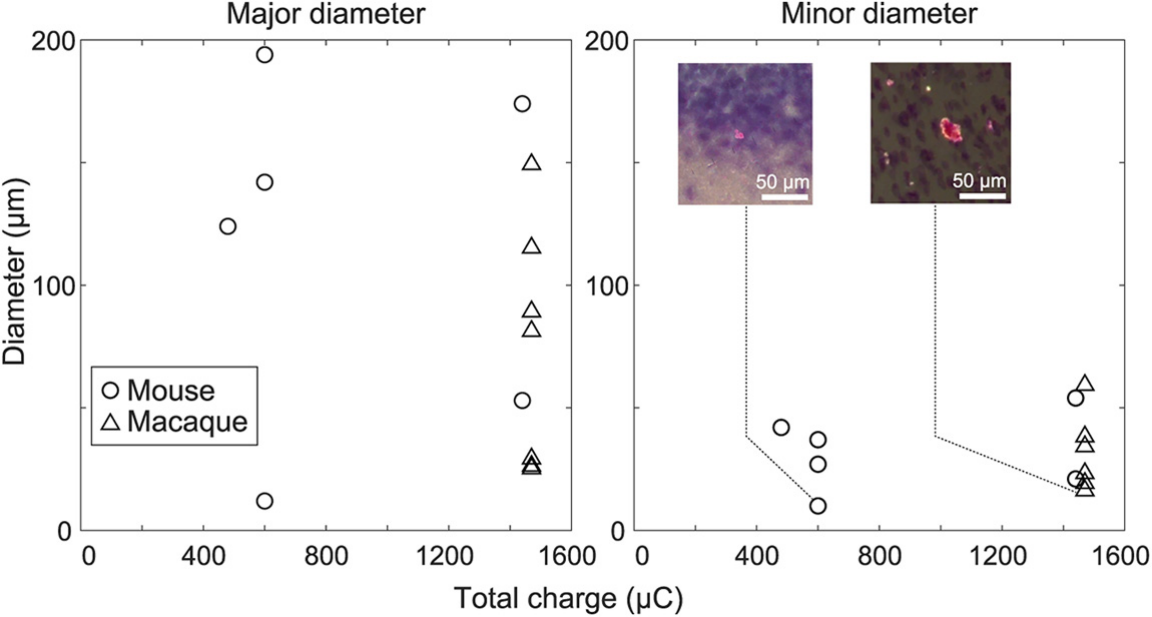
The relationship between the size of marking points (ordinates) and total charge applied to the electrolytic deposition (abscissa). The plots are separated by the major and minor diameters of the markings (left and right, respectively). Please note that the analysis excludes the markings of powdery line (Fig. 3*C*).

The average size of the marking was smaller than that of conventional marking methods. On averaging, the marking size was found to be 32 and 116 μm in minor and major diameters in the mouse experiment, and 30 and 73 μm in the macaque experiment, respectively. The smallest marking size measured <20 μm, corresponding to the size of neuronal soma (Fig. 4). As the aperture length of a 1-MΩ tungsten electrode is ∼10–20 μm (Hubel, 1957; Levick, 1972; Sugiyama et al., 1994), this minimum marking size is sufficient to identify the recording site for the extracellular recording method. In addition, the marking position is unlikely to shift after marking. This is evident from the coincidence of the position with simultaneous marking using fluorescent dye (Fig. 2) and the coincidence of the position with minor electrical lesions (Fig. 3).

Identification of the markings was provided by dark-field observation after Nissl staining. The very bright markings facilitate detection even in low magnification images, which is particularly crucial for smaller markings that can only be distinguished at high magnification. With strong reflected light in dark-field observation, detection is possible at low magnification and with a wide field of view, thereby reducing the time required to search for markings.

In addition to ease of detection, it is crucial to differentiate the markings from other artifacts. For instance, dust particles are often observed as dark dots under bright-field observation, which can make it challenging to differentiate between markings and artifacts because both artifacts and markings appear as dark dots in bright-field observation. In dark-field observation, artifacts also appear as dark dots, whereas our markings appear bright red, enabling a clear distinction between the two. We observed no changes in the appearance of artifacts between bright-field and dark-field observations in the brain tissue, indicating the high identifiability of this marking method.

The tissue damage resulting from this marking technique was found to be minimal. It is known that the use of DC for lesion marking is highly invasive to the tissue, as evidenced by the fact that applying 5 μA for 5 s produces regions with a diameter of 100–200 μm (Hubel, 1959; Koyano et al., 2011). In contrast, AC current is believed to be less damaging (Lilly et al., 1955; Murasugi et al., 1993; Tehovnik, 1996; Fung et al., 1998; Cohen and Newsome, 2004; Murphey and Maunsell, 2007; Clark et al., 2011; Cicmil and Krug, 2015). For instance, bipolar pulses are considered less invasive in electrical stimulation experiments, and microstimulation with biphasic pulses is commonly used to modulate neural activity (Lilly et al., 1955; Salzman et al., 1990, 1992; Murasugi et al., 1993; Tehovnik, 1996; Fung et al., 1998; Cohen and Newsome, 2004; Clark et al., 2011; Cicmil and Krug, 2015). The current used in our technique (40–50 μA) was consistent with that used in other electrical stimulation studies (10–100 μA) and is considered to be minimally invasive. The low invasiveness is of great importance for two reasons. First, preserving the tissue allows for correlation of the recording site with the anatomic structure. Second, recording from nearby the site becomes possible even after marking, which is highly beneficial in chronic experiments where neuronal activity needs to be measured repeatedly from same area over a long period of time.

The markings remained *in vivo* for at least two years. This result indicates that the new marking technique has low long-term toxicity. While it is unclear how the markings may change over time, their ability to maintain sufficient performance for at least two years is promising. This long-term stability would be particularly valuable for chronic experiments.

The new marking technique offers a significant advantage in terms of low implementation costs. Tungsten electrodes are widely used because of their cost-effectiveness. Deposition of tungsten oxide can be produced by applying a bipolar pulse to a tungsten electrode, with its electrical conditions being similar to those used for general microstimulation experiments. Consequently, an electrical stimulator commonly used in electrophysiology experiments can be used. The Nissl staining technique with cresyl violet is widely used and does not require additional staining for our marking. Additionally, the resulting markings can be observed through bright-field or dark-field observation using a conventional optical microscope. The electrode, stimulator, staining, and observation methods described above are all easily implementable, which is an advantage of this method.

### Limitations

In our experiments, there was a 35% probability of tungsten oxide not being released from the electrode, which could only be determined through postmortem slice observation. In order to increase the success rate, electrical parameters must be taken into account. First, a sufficient voltage must be applied as electrochemical reactions typically have a threshold voltage (Ibe et al., 1990). The current value used in this study (40 μA) corresponds to several tens of volts at an electrode with an impedance of ∼1 MΩ, and actual measurements showed that a maximum of ∼17 V was achieved during the pulse period. This is a sufficiently high voltage. Second, the total amount of charge applied can be increased by extending the time of current application. Although the diameter of the markings was not correlated with the total charge applied in our study (Fig. 4), the amount of charge applied is thought to correspond to the amount of tungsten oxide generated. Therefore, it is expected that increasing the charge will lead to an increase in the amount of tungsten oxide, which will make the marking process more successful. However, an excessive amount of charge may result in coarse marking and potential damage to the living tissue. This creates a trade-off between the probability of success and the accuracy of marking. Proper parameter selection is therefore required depending on the research objective.

**Figure 5.**
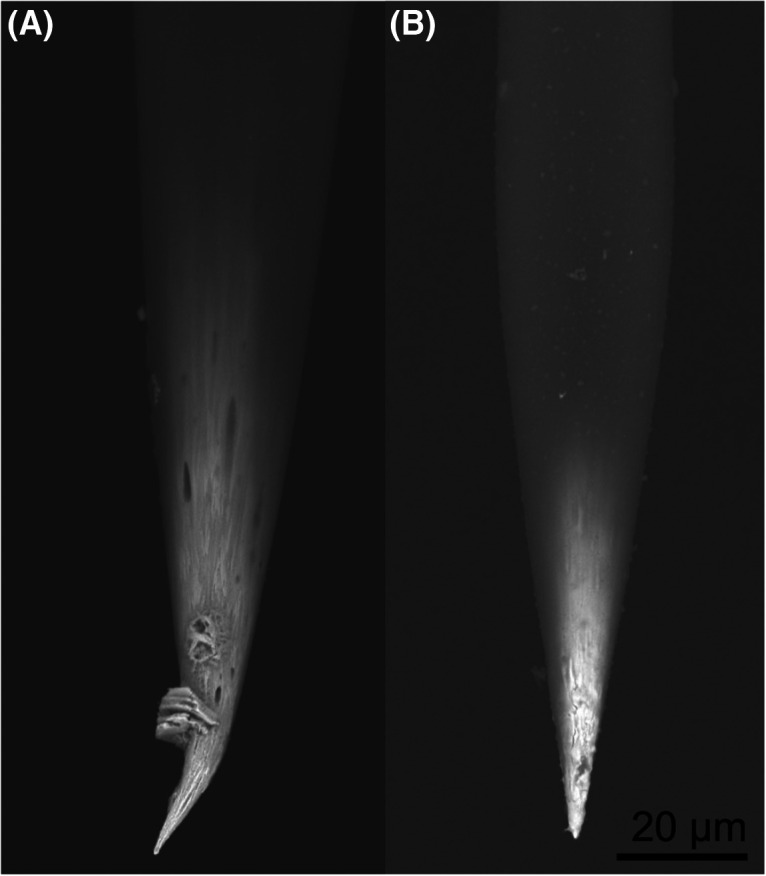
The tips of two representative tungsten microelectrodes observed using a scanning electron microscope (Miniscope TM1000, Hitachi). ***A*** and ***B*** are unused electrodes from the same lot.

Even successful cases, the markings exhibited variability in size and shape, which corresponded the precision of the marking. This variability could be attributed to individual variances of the electrodes. As demonstrated in the mouse experiments, the size of the markings varied even when the same electrical parameters were used (Fig. 2). The variation in marking size may be because of several reasons. First, it may be attributed to variations in the shape and microstructure of the tip of electrodes. To investigate this, we examined two unused electrodes from the same lot using a scanning electron microscope (Fig. 5). Observed image revealed that the surfaces of the electrode tip were uneven on the scale of 1 μm and displayed different patterns on each electrode. The morphology of the electrode surface could impact the shape of the oxide to be deposited, as well as its successful detachment from the electrode. Second, it could be affected by the tip aperture shape of the insulating coating. Tungsten electrodes are produced by a tungsten wire with an insulation coating, and then stripping the insulation coating at the tip (Hubel, 1957; Sugiyama et al., 1994). Although the aperture size at the electrode tip is roughly homogenized by impedance measurements, the shape of the aperture may vary. In the future, if it becomes feasible to predict how tungsten oxide is generated based on the electrode and aperture shape, it may be possible to increase the success rate by selecting the appropriate electrode for marking.

The electrodes could be reused multiple times, depending on the total charge used. The marking technique employs an oxidation reaction, causing corrosion of the tip and electrode damage. This was shown by minor tip rounding and decreased impedance after the marking. We also noticed that isolation performance of the single-unit recording declined after the marking. However, multiunit recording remained viable, with marking repeatable at least up to three times.

Since tungsten oxide is a solid particle, it may deteriorate its positional accuracy in two possible ways. First, tungsten oxide that adheres to the electrode surface after marking may spill onto the track because of electrode movement. This can introduce noise and reduce the positional accuracy of the marking. Second, during brain slice preparation, the tungsten oxide may be displaced by the cutting blade and diffuse into the surrounding tissue, or the markings themselves may fall out of the tissue during manipulation of the slice. To alleviate this issue, increasing the thickness of the brain slice could be beneficial as it can decrease the probability of the blade striking the marking and can increase the likelihood of the marking point remaining within the tissue.

Tungsten oxide was observed on the penetration path of the tungsten electrode even in the absence of electrical stimulation, likely resulting from natural oxidation of the electrode surface. In the experiment on the monkey brain slice, such unintended tungsten oxide was found abundantly on the surface of the brain where no electrical stimulation had been applied. This may have been because of stress on the electrode at the site of brain puncture, which could have caused the oxide to detach and contaminate the surroundings. Unintentional tungsten oxide can introduce noise to the marking, which is particularly problematic when marking is desired near the brain puncture site. A potential solution to this issue is to mechanically polish the tip before electrode puncture or to apply an anode pulse beforehand to remove the oxide at the electrode interface.

Our marking method is only applicable for tungsten electrodes. There are several other materials that are commonly used for metal electrodes. For instance, in the case of an alloy containing iron, metal ions would be deposited if a biphasic current was applied (Fung et al., 1998). With the platinum electrodes, no metal oxidation reaction occurs under the current application (Robblee and Sweeney, 1996). Therefore, considering that contemporary multichannel electrode tips are made of platinum alloy, our marking method cannot be directly applied to these modern electrodes.

### Tungsten oxide

The substance deposited by the markings is considered to be tungsten oxide (Ibe et al., 1990). The markings were black or reddish brown before Nissl staining (Fig. 1*B*). This color is related to the chemical composition of the material. Tungsten oxide is a negatively charged ion that binds to sodium or hydrogen. It is known that the ratio of sodium to hydrogen can significantly affect the apparent color (Tegg et al., 2017). The reddish brown color corresponds to sodium tungsten bronze Na_x_WO_3_ (x = 0.75). While the marking points appeared brown in bright-field observation (Figs. 2*G*,*K*, 3*D*), they appeared reddish pink in dark-field observation after Nissl staining (Figs. 2*H*,*L*, 3*E*). The red-pink color is not the color of tungsten oxide, but is similar to that of cresyl violet. Cresyl violet is a basic dye (Carter and Shieh, 2015) and may have labeled negatively charged tungsten oxide (WO_3_^−^). Based on this information, it can be inferred that the marking points consist of tungsten oxide with cresyl violet attached to it.

Pabst described a technique for observing “tungsten blue” (possibly H_x_WO_3_; x = 0.1−0.5) by reducing tungsten oxide with osmic acid (Pabst, 1973). This method was based on the fact that the color of tungsten oxide changes significantly with pH. However, simultaneous staining of both tungsten oxide and cells is challenging because of the pH dependency. Many staining techniques, including Nissl staining, are conducted in neutral solutions (pH ∼7), which makes it difficult to perform tungsten blue staining at the same time as cell staining. In contrast, our method employing Nissl staining can visualize both tungsten oxide and cells. Thus, our method outperforms Pabst’s tungsten blue staining method.

### Future direction

Electroetching of tungsten needles is widely used to produce electrodes for electrophysiology experiments. Although electroetching is well known in the context of needle fabrication, little attention has been paid to the oxides and the debris produced during the etching process. For example, Histed et al., demonstrated in their supplementary the deposition of tungsten ion caused by electrostimulation with a tungsten electrode (Histed et al., 2009). Similarly, despite the large number of studies in which electrical stimulation was performed with tungsten electrodes (Salzman et al., 1992; Murasugi et al., 1993; Cicmil and Krug, 2015), some researchers might have noticed the deposition if they had observed slices at the stimulation site. However, to our knowledge, no study has indicated that such a deposition can be used as a marking. If the tissue slides of electrically stimulated tissue are preserved, then the markings could be used to indicate the exact site of electrical stimulation. Pabst’s method was not widely employed, probably because of issues related to invasiveness and identifiability. However, the method described in this study overcomes these challenges and enables the inexpensive and simple mapping of brain functions to anatomic structures.

## Synthesis

Reviewing Editor: Matthew Grubb, King’s College London Decisions are customarily a result of the Reviewing Editor and the peer reviewers coming together and discussing their recommendations until a consensus is reached. When revisions are invited, a fact-based synthesis statement explaining their decision and outlining what is needed to prepare a revision will be listed below. The following reviewer(s) agreed to reveal their identity: Paul Martin. Note: If this manuscript was transferred from JNeurosci and a decision was made to accept the manuscript without peer review, a brief statement to this effect will instead be what is listed below.

Reviewer 1:

Oikawa and colleagues report a simple but significant modification to time-honored electrolytic lesion methods to mark recording electrode tip location. The modification is to use biphasic current pulses. The paper is clear and well documented and the limitations of current methods accurately portrayed. It could be argued that a methods-based journal would be a better place to publish this advance but the general significance for single-cell extracellular recording is high. Although the exact electrochemistry of the reaction is not the issue of study it seems to be clearly and logically explained. My only gripe is that I cannot find the temporal waveform of stimulation i.e the duration of positive and negative phases of stimulation. This needs to be stated. It might also be helpful to cite Levick WR (1972) Another tungsten microelectrode. Med Biol Eng 10:510-515: These electrodes are maddeningly fragile but offer recording benefits that some readers may find useful.

Minor.

11 and elsewhere / proposes / describes /

49 / highly // [suggest delete word]

Reviewer 2:

The paper, “A fine-scale and minimally invasive marking method for use with conventional tungsten microelectrodes” by Oikawa et al., introduces a new method for marking electrophysiology recording sites. By utilizing a biphasic current pulse sequence, tungsten oxide is deposited at the marking site, which is easily distinguished via dark-field microscopy. This simple and novel technique has potential widespread applicability in many neuroscience labs conducting electrophysiology.

The paper is well written and presents an advancement over existing methods for localizing microelectrode recording sites, contributing positively to neuroscience. Notably, the use of biphasic current addresses difficulties described in a previous work by Pabst (1973), which often required manual reversal of current polarity. The biphasic current also potentially reduces tissue damage. The easily detectable tungsten oxide mark, with its high contrast under dark-field microscopy, is a significant advantage. Furthermore, the mark remains visible for more than two years, facilitating its use in chronic experiments.

I have a few suggestions and comments to enhance clarity and further strengthen the paper as outlined below.

Major comment:

1. Quantification

The authors provided measurements for the size of the marks but only for a few examples showcased in the figures (50x100 um/ 10x30 um for Fig. 2 and 100x30 um for Fig. 3). Additionally, while the authors note that the marking size ranges between 10 to 100 um (Line 181), it’s unclear whether this is relevant to the mouse experiment, the macaque experiment, or both. Could the authors provide more detailed data, such as the distribution of minor and major diameter and their mean and standard deviation? Without such comprehensive quantification, it’s challenging to evaluate the claim that these marker sizes are smaller than those of previous work (Line 199), which is a major assertion in this paper. More detailed information could significantly support this claim.

Minor comments:

1. Lines 34-37, “Multi-electrode arrays ... can cause significant brain damage”; “In contrast, single-unit recording can record ... with less damage”: Could you add reference(s) that quantified significant difference of brain damage with these electrodes? This would strengthen this statement.

2. Line 37, “single-unit recording”: Please clarify whether this refers to a single-channel electrode. Multi-electrode arrays can also isolate single units.

3. Lines 61-62, “insufficient to accurately localize the site at the cellular size of approximately 10 μm”. The mark size in the current study is larger than 10 μm. Please better define the scope of this paper and the target marking size.

4. Lines 62-65, “Although the size of lesion marking and iron ion marking ... can cause tissue damage and obscure anatomical structures.”: I recommend discussing the method presented by Fung et al. (1998), who used a biphasic current in their iron marking method to minimize tissue damage. Their marks remained visible with MRI for at least two months, and were also histologically detectable. This could offer a relevant comparison to the current work.

5. Line 73, “such as layers of the lateral geniculate nucleus, superior colliculus, and pulvinar.”: As far as I’m aware, there’s no known anatomical layer structure in the pulvinar, and the pulvinar is much larger than 100 μm.

6. Lines 182-183, “the size of the aperture of a tungsten electrode is approximately 10-20 μm”: Please specify if this is referring to the diameter or the length of the aperture.

7. Lines 183-184, “10 μm is sufficient to identify the recording site for the extracellular recording method”.: The size of 10 μm represents the upper limit (smallest size) of the current method. Please consider discussing based on the average size or lower limit (100 um) of the marks.

8. Lines 233-234, “As demonstrated in the mouse experiments, the size of the markings varied even when the same electrical parameters were used (Fig. 3).”: Please correct the reference to the mouse experiments in Fig. 2, not Fig. 3.

9. Lines 236-242, “To investigate this, ... the aperture may vary.” Could you provide photographs and electron microscope images of the tungsten electrodes used in this study? This additional information would be valuable to researchers attempting to use this method.

10. With the recent significant advancements in electrode technology, as the authors mentioned in the first sentence of the significant statement and the first paragraph of the Introduction, is there potential to incorporate this marking method with new types of electrodes? This could be an interesting point for discussion.

Reference:

Fung SH, Burstein D, Born RT. In vivo microelectrode track reconstruction using magnetic resonance imaging. J Neurosci Methods. 1998 Apr 30;80(2):215-24.

## Author Response

Reviewer 1:

Oikawa and colleagues report a simple but significant modification to time-honored electrolytic lesion methods to mark recording electrode tip location. The modification is to use biphasic current pulses. The paper is clear and well documented and the limitations of current methods accurately portrayed. It could be argued that a methods-based journal would be a better place to publish this advance but the general significance for single-cell extracellular recording is high. Although the exact electrochemistry of the reaction is not the issue of study it seems to be clearly and logically explained. My only gripe is that I cannot find the temporal waveform of stimulation i.e the duration of positive and negative phases of stimulation. This needs to be stated. It might also be helpful to cite Levick WR (1972) Another tungsten microelectrode. Med Biol Eng 10:510-515: These electrodes are maddeningly fragile but offer recording benefits that some readers may find useful.

We added the temporal waveform of stimulation in figure 2D and figure 3A. We agree Levick (1972) is helpful thus the citation is included in L180 and 187.

Minor.

11 and elsewhere / proposes / describes /

Modified as suggested. (L17, 74, 178, 293, 309)

49 / highly // [suggest delete word]

Modified as suggested. (L47)

Reviewer 2:

The paper, “A fine-scale and minimally invasive marking method for use with conventional tungsten microelectrodes” by Oikawa et al., introduces a new method for marking electrophysiology recording sites. By utilizing a biphasic current pulse sequence, tungsten oxide is deposited at the marking site, which is easily distinguished via dark-field microscopy. This simple and novel technique has potential widespread applicability in many neuroscience labs conducting electrophysiology.

The paper is well written and presents an advancement over existing methods for localizing microelectrode recording sites, contributing positively to neuroscience. Notably, the use of biphasic current addresses difficulties described in a previous work by Pabst (1973), which often required manual reversal of current polarity. The biphasic current also potentially reduces tissue damage. The easily detectable tungsten oxide mark, with its high contrast under dark-field microscopy, is a significant advantage. Furthermore, the mark remains visible for more than two years, facilitating its use in chronic experiments.

I have a few suggestions and comments to enhance clarity and further strengthen the paper as outlined below.

Major comment:

1. Quantification

The authors provided measurements for the size of the marks but only for a few examples showcased in the figures (50x100 um/ 10x30 um for Fig. 2 and 100x30 um for Fig. 3). Additionally, while the authors note that the marking size ranges between 10 to 100 um (Line 181), it’s unclear whether this is relevant to the mouse experiment, the macaque experiment, or both. Could the authors provide more detailed data, such as the distribution of minor and major diameter and their mean and standard deviation? Without such comprehensive quantification, it’s challenging to evaluate the claim that these marker sizes are smaller than those of previous work (Line 199), which is a major assertion in this paper. More detailed information could significantly support this claim.

We added the distribution of the size of the mark in figure 4. Quantitative description of the mark was added for each example (L120, 136, 158, 165, 184).

Minor comments:

1. Lines 34-37, “Multi-electrode arrays ... can cause significant brain damage”; “In contrast, single-unit recording can record ... with less damage”: Could you add reference(s) that quantified significant difference of brain damage with these electrodes? This would strengthen this statement.

We added the citation which discussed the correlation between electrode size and the extent of brain damage.

L35: In contrast, single-channel electrodes generally possess a small diameter, enabling the recording of single-unit activities from deep brain regions with less damage18 and at a lower cost.

*18 Szarowski et al. Brain responses to micro-machined silicon devices. Brain Res. 983, 23-35 (2003).

2. Line 37, “single-unit recording”: Please clarify whether this refers to a single-channel electrode. Multi-electrode arrays can also isolate single units.

“Single-channel” is correct. We modified the description. (L35)

3. Lines 61-62, “insufficient to accurately localize the site at the cellular size of approximately 10 μm”. The mark size in the current study is larger than 10 μm. Please better define the scope of this paper and the target marking size.

We modified the scope of the study.

L59: The existing marking methods typically have a width of around 100 μm, which is insufficient to accurately localize the site smaller than 100 μm, such as suborganizations within small nuclei.

4. Lines 62-65, “Although the size of lesion marking and iron ion marking ... can cause tissue damage and obscure anatomical structures.”: I recommend discussing the method presented by Fung et al. (1998), who used a biphasic current in their iron marking method to minimize tissue damage. Their marks remained visible with MRI for at least two months, and were also histologically detectable. This could offer a relevant comparison to the current work.

We appreciate your suggestions. Fung et al. showed that damage is reduced by using a biphasic current. This damage reduction is also true for our marking method. On the other hand, they also showed that there is a trade-off between mark persistence and size. This is an important difference with our methodology. We therefore modified ours as follows:

L64: Utilizing biphasic currents instead of DC during iron ion markings could reduce damage, however there is still a tradeoff between the persistence of the marking and their size42.

*43 Fung et al (1998)

The citations were also added L208, L210 and L280.

5. Line 73, “such as layers of the lateral geniculate nucleus, superior colliculus, and pulvinar.”: As far as I’m aware, there’s no known anatomical layer structure in the pulvinar, and the pulvinar is much larger than 100 μm.

We removed “and pulvinar” (L73).

6. Lines 182-183, “the size of the aperture of a tungsten electrode is approximately 10-20 μm”: Please specify if this is referring to the diameter or the length of the aperture.

The length. We modified description as follows:

L187: As the aperture length of a 1-MΩ tungsten electrode is approximately 10-20 μm56,58,59,

7. Lines 183-184, “10 μm is sufficient to identify the recording site for the extracellular recording method”.: The size of 10 μm represents the upper limit (smallest size) of the current method. Please consider discussing based on the average size or lower limit (100 um) of the marks.

We modified the discussion based on the comparison between average size of the mark and conventional methods.

L184: The average size of the marking was smaller than that of conventional marking methods. On averaging, the marking size was found to be 32 μm and 116 μm in minor and major diameters in the mouse experiment, and 30 μm and 73 μm in the macaque experiment, respectively. The smallest marking size measured < 20 μm, corresponding to the size of neuronal soma (Fig. 4).

8. Lines 233-234, “As demonstrated in the mouse experiments, the size of the markings varied even when the same electrical parameters were used (Fig. 3).”: Please correct the reference to the mouse experiments in Fig. 2, not Fig. 3.

We modified as suggested. (L244)

9. Lines 236-242, “To investigate this, ... the aperture may vary.” Could you provide photographs and electron microscope images of the tungsten electrodes used in this study? This additional information would be valuable to researchers attempting to use this method.

We added the SEM images of representative electrodes (Fig 5). They have different texture on their tip surface.

L246: To investigate this, we examined two unused electrodes from the same lot using a scanning electron microscope (Fig. 5).

10. With the recent significant advancements in electrode technology, as the authors mentioned in the first sentence of the significant statement and the first paragraph of the Introduction, is there potential to incorporate this marking method with new types of electrodes? This could be an interesting point for discussion.

Thank you for giving us an important perspective. Unfortunately, this method is dependent on the electrode material, so it seems difficult to apply it to modern electrodes. This is because many modern multichannel electrodes are made of platinum alloys, which are not prone to metal oxidation reactions. We have described this point in one of the paragraphs of the discussion.

L278: Our marking method is only applicable for tungsten electrodes. There are several other materials that are commonly used for metal electrodes. For instance, in the case of an alloy containing iron, metal ions would be deposited if a biphasic current was applied42. With the platinum electrodes, no metal oxidation reaction occurs under the current application50. Therefore, considering that contemporary multi-channel electrode tips are made of platinum alloy, our marking method cannot be directly applied to these modern electrodes.
